# One-Hour Post-Load Glucose Is Associated with Multisystem Complications in People Living with Obesity

**DOI:** 10.3390/biom16020268

**Published:** 2026-02-09

**Authors:** Ioanna Mixaki, Michalis G. Prokopakis, Georgios Dimakopoulos, Theodosios D. Filippatos, Kalliopi Kotsa, Theocharis Koufakis

**Affiliations:** 1 Medical School, Aristotle University, 54124 Thessaloniki, Greece; jmixaki@hotmail.com; 2 Department of Internal Medicine, School of Medicine, University of Crete, 71003 Heraklion, Greece; micprokopakis@gmail.com (M.G.P.); filtheo@uoc.gr (T.D.F.); 3 BIOSTATS, Epirus Science and Technology Park Campus, University of Ioannina, 45110 Ioannina, Greece; info@biostats.gr; 4 Division of Endocrinology and Metabolism and Diabetes Center, First Department of Internal Medicine, Medical School, Aristotle University of Thessaloniki, AHEPA University Hospital, 54636 Thessaloniki, Greece; kkalli@auth.gr; 5 Second Propaedeutic Department of Internal Medicine, Hippokration General Hospital, Aristotle University of Thessaloniki, 54642 Thessaloniki, Greece

**Keywords:** obesity, oral glucose tolerance test, one-hour glucose, obesity-related complications, insulin resistance

## Abstract

**Background:** Obesity is associated with a broad range of complications that frequently develop before the onset of type 2 diabetes mellitus (T2DM). Identifying early metabolic markers associated with such complications is essential for improving risk stratification and supporting complication-driven therapeutic strategies. Plasma glucose measured at 1 h during the oral glucose tolerance test (OGTT) has emerged as a sensitive marker of early dysglycemia and adverse cardiometabolic outcomes, but its relationship with established obesity-related complications in individuals without diabetes remains incompletely characterized. We aimed to investigate the association between 1 h post-load plasma glucose levels during OGTT and obesity-related complications in adults living with obesity without T2DM. **Methods:** This observational cross-sectional study included 47 adults with obesity evaluated during their first visit to obesity clinics. Individuals with T2DM or prior use of anti-obesity pharmacotherapy were excluded. All participants underwent a standard 75-g OGTT with plasma glucose and insulin measurements at fasting, 1 h, and 2 h. Obesity-related complications were recorded retrospectively through structured questionnaires, clinical assessment, and medical record review. Between-group comparisons were performed using non-parametric tests, and repeated OGTT measurements were analyzed using non-parametric longitudinal models. **Results:** Higher 1 h post-load glucose levels were observed in participants with arterial hypertension (*p* < 0.001), dyslipidemia (*p* = 0.020), metabolic dysfunction-associated steatotic liver disease (*p* = 0.005), impaired glucose tolerance (*p* = 0.027), obesity hypoventilation syndrome (*p* = 0.039), urinary incontinence (*p* = 0.038), and chronic kidney disease (*p* = 0.048). In most comparisons, 1 h post-load glucose demonstrated stronger discriminatory capacity than fasting or 2 h glucose values. Insulin levels increased markedly after glucose loading in all participants, reflecting generalized insulin resistance, but showed limited ability to discriminate between complication phenotypes. **Conclusions:** In people living with obesity without T2DM, elevated 1 h post-load plasma glucose during OGTT is consistently associated with multisystem obesity-related complications. These findings support the clinical relevance of 1 h post-load glucose as an integrated marker of early metabolic and systemic burden that may inform complication-driven risk stratification in obesity. Due to the observational study design, causality cannot be inferred.

## 1. Introduction

Obesity has reached epidemic proportions globally and represents one of the most pressing public health challenges of contemporary medicine [1]. Its prevalence continues to rise across all age groups and geographic regions, contributing substantially to global morbidity, mortality, and healthcare expenditure. Importantly, obesity is no longer regarded solely as excessive fat accumulation but rather as a complex, chronic, and multisystem disease characterized by profound metabolic, hormonal, inflammatory, and structural alterations [2]. These disturbances underlie a wide spectrum of obesity-related complications involving the cardiovascular, hepatic, renal, respiratory, musculoskeletal, and genitourinary systems, as well as functional impairment and reduced quality of life [3].

Although type 2 diabetes mellitus (T2DM) is one of the most recognized metabolic consequences of obesity, many obesity-related complications develop independently of diabetes and often precede its onset by several years [4]. Individuals living with obesity may therefore experience significant organ dysfunction despite glucose values that do not meet diagnostic thresholds for diabetes [5]. This observation highlights important limitations of traditional glycemic markers and underscores the need for more sensitive indicators capable of identifying individuals at increased risk before irreversible metabolic and organ damage occurs [6].

The oral glucose tolerance test (OGTT) remains a cornerstone in the assessment of glucose metabolism [7]. Historically, clinical interpretation has focused on fasting and 2 h post-load glucose values. However, these measures provide only a partial representation of postprandial glucose handling and may fail to detect early abnormalities in glucose excursion [8]. Over the past decade, increasing attention has been directed toward plasma glucose measured at 1 h during OGTT. Elevated 1 h post-load glucose has been shown to reflect impaired insulin sensitivity, β-cell dysfunction, altered hepatic glucose handling, and reduced metabolic flexibility [9]. Importantly, multiple longitudinal studies have demonstrated that 1 h post-load glucose predicts progression to T2DM, cardiovascular events, microvascular complications, and all-cause mortality, often more reliably than fasting or 2 h glucose values [10]. However, obesity-related complications arise from complex interactions among multiple cardiometabolic risk factors, including dyslipidemia, hypertension, insulin resistance, inflammation, and lifestyle-related exposures, and the independent contribution of post-load glycemia remains incompletely defined.

Despite this growing body of evidence, the clinical significance of 1 h post-load glucose in relation to obesity-related complications remains insufficiently explored. In particular, it is unclear whether elevated 1 h post-load glucose is associated with the presence of established complications in individuals living with obesity who do not yet have diabetes, rather than merely predicting future metabolic deterioration. Addressing this gap is particularly relevant in the context of emerging obesity management frameworks that emphasize complication-driven treatment strategies [11]. Therefore, the aim of the present study was to examine associations between 1 h post-load plasma glucose values during OGTT and a broad spectrum of obesity-related complications in adults living with obesity without T2DM.

## 2. Methods

### 2.1. Study Design, Setting, and Participants

This was an observational, cross-sectional study conducted in adults living with obesity attending specialized obesity outpatient clinics at a tertiary-care university hospital. Participants were evaluated at their first clinical visit between September and December 2024, prior to the initiation of any structured dietary, lifestyle, pharmacological, or surgical weight-loss intervention. Recruitment was based on consecutive referrals to the obesity clinics during routine clinical practice within this predefined time frame, in order to minimize selection bias and enhance external validity.

Obesity was defined according to body mass index (BMI) criteria (≥30 kg/m^2^) [12]. Eligible participants were adults with obesity who underwent a comprehensive baseline evaluation, including detailed medical history, physical examination, anthropometric assessment, biochemical testing, and an OGTT. Individuals with previously diagnosed or newly identified T2DM, as well as those receiving anti-obesity medications or glucose-lowering therapies, were excluded to minimize confounding effects on glucose metabolism and insulin dynamics.

Owing to the exploratory, hypothesis-generating nature of the study and the consecutive recruitment within a predefined time window, no a priori sample size calculation was performed.

### 2.2. Assessment and Definition of Obesity-Related Complications

Obesity-related complications were recorded retrospectively at baseline using a structured medical questionnaire administered during the first visit, combined with clinical evaluation and review of available medical records. Complications were identified based on standard clinical diagnostic criteria, as documented by treating physicians in the medical records. The assessment of complications was clinically driven rather than based on systematic, protocol-mandated screening, and participants did not undergo uniform diagnostic testing for all conditions solely for the purposes of the study.

The assessment encompassed a broad range of obesity-related conditions, including cardiometabolic complications (such as arterial hypertension, dyslipidemia, atherosclerotic cardiovascular disease [ASCVD], and impaired glucose tolerance [IGT]), hepatic disease (metabolic dysfunction-associated steatotic liver disease [MASLD]), renal disease (chronic kidney disease [CKD]), respiratory disorders (obesity hypoventilation syndrome [OHS] and chronic obstructive pulmonary disease [COPD]), genitourinary conditions (urinary incontinence), and endocrine disorders (polycystic ovary syndrome [PCOS]). For example, arterial hypertension was defined by a prior clinical diagnosis or use of antihypertensive medication, dyslipidemia by a documented diagnosis or lipid-lowering therapy, and impaired glucose tolerance according to OGTT criteria [13]. The presence or absence of each complication was recorded at the time of the initial clinical evaluation. OHS and COPD were analyzed as a single composite respiratory complication due to limited numbers in each category.

### 2.3. Anthropometric Measurements and Laboratory Assessment

Anthropometric measurements were obtained using standardized procedures by trained healthcare personnel. Body weight and height were measured with participants wearing light clothing and no shoes, and BMI was calculated as weight in kilograms divided by height in meters squared. Waist and hip circumferences were measured using a flexible tape at standardized anatomical landmarks, and indices of central adiposity were derived accordingly.

Blood samples were collected in the morning after an overnight fast of at least 8–12 h, during which participants were instructed to abstain from food, caloric beverages, smoking, and vigorous physical activity. Participants were clinically stable at the time of testing, with no evidence of acute illness, and had stable body weight in the period preceding evaluation.

All participants underwent a standard 75 g OGTT according to established clinical protocols. Following fasting blood sampling for plasma glucose and insulin measurements, participants ingested 75 g of anhydrous glucose dissolved in water within a standardized time period. Additional blood samples were obtained at 1 h and 2 h after glucose ingestion. Participants remained seated throughout the test and refrained from physical activity, smoking, or food intake during the testing period.

Plasma glucose concentrations were measured in venous samples using enzymatic methods, and insulin concentrations were determined using standardized immunoassays, as part of routine laboratory procedures at the university hospital laboratory. Routine biochemical analyses also included lipid profile, liver enzymes, renal function indices, inflammatory markers (C-reactive protein [CRP]), and hormonal parameters.

### 2.4. Statistical Analysis

Data were described using means and standard deviations for continuous variables and absolute numbers and percentages for categorical variables, including recorded obesity-related complications. Between-group comparisons were performed using non-parametric Mann–Whitney U tests, given the non-normal distribution of several variables and the modest sample size. Repeated measurements of plasma glucose and insulin during OGTT were analyzed using non-parametric longitudinal methods with an F1-LD-F1 design, implemented through the nparLD package in R (4.5.1). This approach provided estimates of statistical significance for within-group effects, between-group effects, and interaction terms. When overall statistical significance was observed, pairwise comparisons were conducted using the Holm–Bonferroni procedure to adjust for multiple testing and control inflation of the type I error rate. All statistically significant results are reported using the adjusted significance level. Analyses were conducted using complete-case data; no imputation of missing values was performed. All statistical analyses were performed using RStudio version 2024.12.1 for Windows. No multivariable adjustment was performed, as obesity-related complications were recorded retrospectively and not within a predefined causal or temporal analytical framework. Under these conditions, key covariates (e.g., BMI, hypertension, dyslipidemia) may act as confounders, mediators, or consequences of post-load dysglycemia, rendering multivariable modeling susceptible to overadjustment or collider bias and potentially misleading estimates of independent effects [14,15].

### 2.5. Ethical Considerations

The study was conducted in accordance with the principles of the Declaration of Helsinki. Written informed consent was obtained from all participants prior to inclusion. All data were collected as part of routine clinical care, anonymized before analysis, and the study protocol was approved by the ethics committee of the Aristotle University of Thessaloniki (approval code: 6; approval date: 10 August 2024).

## 3. Results

### 3.1. Baseline Anthropometric Characteristics and OGTT Metabolic Responses

The study population comprised 47 adults living with obesity, with a mean age of 47.53 ± 11.29 years. Mean body weight was 106.73 ± 23.59 kg, and mean BMI was 37.01 ± 7.14 kg/m^2^. Measures of central adiposity were markedly elevated, including a mean waist circumference of 113.38 ± 16.80 cm and a mean waist-to-height ratio of 0.67 ± 0.11.

Mean fasting plasma glucose was 102.10 ± 20.00 mg/dL. During OGTT, plasma glucose concentrations increased to 156.90 ± 56.20 mg/dL at 1 h and declined to 108.50 ± 45.20 mg/dL at 2 h. Insulin concentrations increased substantially following glucose ingestion, from 24.30 ± 27.80 μIU/mL at fasting to 125.30 ± 153.90 μIU/mL at 1 h, remaining elevated at 75.00 ± 59.80 μIU/mL at 2 h.

Pairwise comparisons confirmed statistically significant differences in insulin concentrations between fasting and 1 h and between fasting and 2 h (both *p* < 0.001). In contrast, the difference between 1 h and 2 h post-load insulin concentrations was not statistically significant in the overall cohort after adjustment for multiple comparisons, indicating that peak insulin concentrations occurred at approximately 1 h after glucose ingestion.

Non-parametric longitudinal analysis of repeated glucose and insulin measurements during OGTT demonstrated a significant within-subject effect of time for both glucose and insulin concentrations (both *p* < 0.001), confirming dynamic post-load responses. Glucose and insulin concentrations increased significantly from fasting to 1 h and declined thereafter. No significant overall time × complication interaction was observed for insulin, indicating a broadly similar temporal pattern across complication groups. Table 1 presents the demographic, anthropometric, metabolic characteristics and prevalence of obesity-related complications in the study population.

### 3.2. Differences in Glucose Values According to Obesity-Related Complications

**Figure 1:**
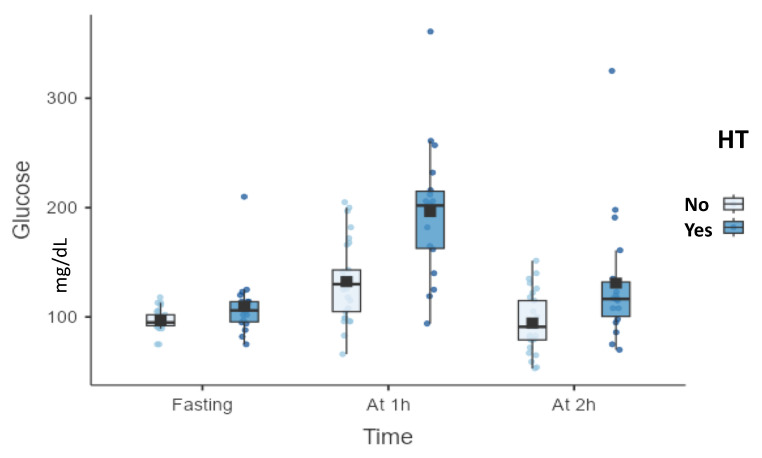
Plasma glucose concentrations during OGTT according to arterial hypertension status. Abbreviations: OGTT, oral glucose tolerance test; HT, arterial hypertension; h, hour(s).

Plasma glucose values during OGTT differed significantly according to the presence of several obesity-related complications, with 1 h post-load glucose consistently demonstrating the strongest and most frequent associations. Participants with arterial hypertension exhibited significantly higher glucose concentrations at all OGTT time points compared with those without hypertension. The difference was most pronounced at 1 h (196.44 ± 61.46 mg/dL vs. 132.38 ± 35.70 mg/dL, *p* < 0.001). Two-hour glucose values were also higher, but the magnitude of difference was smaller than that observed at 1 h (Figure 1).

**Figure 2:**
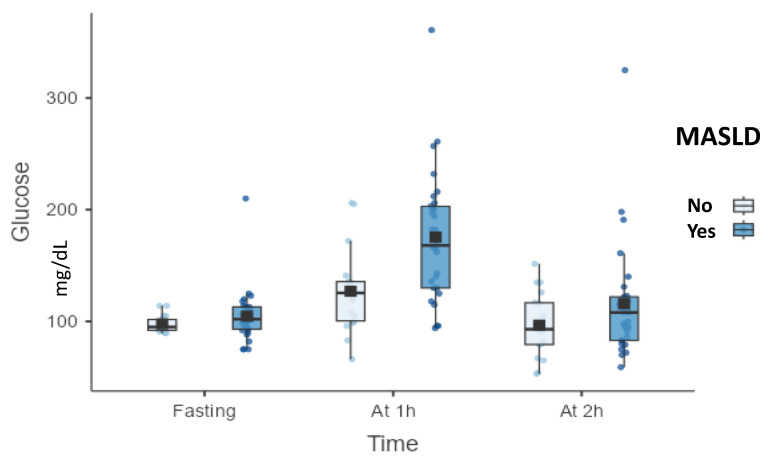
Plasma glucose concentrations during OGTT according to MASLD status. Abbreviations: OGTT, oral glucose tolerance test; MASLD, metabolic dysfunction-associated steatotic liver disease; h, hour(s).

Participants with dyslipidemia demonstrated significantly higher 1 h post-load glucose concentrations compared with those without dyslipidemia (172.03 ± 59.95 mg/dL vs. 130.24 ± 37.35 mg/dL, *p* = 0.020). No statistically significant differences were observed for fasting or 2 h glucose values. Individuals with MASLD exhibited significantly higher 1 h post-load glucose levels compared with those without the condition (*p* = 0.005). Fasting and 2 h glucose values did not differ significantly between groups (Figure 2).

Participants with IGT demonstrated higher plasma glucose concentrations at all OGTT time points compared with those without IGT. The difference was significant for fasting, 1 h (178.60 ± 62.02 mg/dL vs. 140.85 ± 46.43 mg/dL, *p* = 0.027), and 2 h post-load glucose values, with the largest between-group separation observed at 1 h.

Participants with OHS or COPD exhibited significantly higher 1 h post-load glucose concentrations compared with those without respiratory disease (171.27 ± 63.61 mg/dL vs. 139.14 ± 40.22 mg/dL, *p* = 0.039). No significant differences were observed for fasting or 2 h glucose values. Participants with urinary incontinence demonstrated markedly elevated 1 h post-load glucose concentrations compared with those without incontinence (205.25 ± 40.31 mg/dL vs. 152.42 ± 55.72 mg/dL, *p* = 0.038). Fasting and 2 h glucose values did not differ significantly.

Despite the small number of affected participants, individuals with CKD exhibited significantly higher 1 h post-load glucose levels compared with those without renal disease (*p* = 0.048). No significant differences were observed for fasting or 2 h glucose values. No statistically significant differences in fasting, 1 h, or 2 h glucose values were observed between participants with and without PCOS or OSA.

Across the full spectrum of obesity-related complications examined, 1 h post-load glucose was the OGTT-derived glycemic parameter most consistently higher in participants with obesity-related complications, frequently in the absence of corresponding fasting or 2 h glucose differences. Findings related to complications with low prevalence, including CKD and urinary incontinence, should be interpreted cautiously and are presented as exploratory. Differences in 1 h post-load glucose concentrations according to the presence or absence of obesity-related complications are summarized in Table 2.

### 3.3. Insulin Responses According to Complication Status

Insulin concentrations increased significantly over time during OGTT across all participants, reflecting generalized post-load hyperinsulinemia. Between-group comparisons demonstrated limited discriminatory capacity of insulin values across complication categories. Participants with arterial hypertension exhibited significantly higher 2 h insulin concentrations compared with those without hypertension (108.91 ± 74.91 μIU/mL vs. 56.36 ± 40.02 μIU/mL, *p* = 0.017). In participants with urinary incontinence, pairwise comparisons demonstrated a statistically significant difference between 1 h and 2 h post-load insulin concentrations (*p* = 0.045), reflecting a decline from peak insulin levels at 1 h. No statistically significant differences in absolute insulin concentrations were observed between individuals with and without other obesity-related complications. Overall, insulin responses reflected generalized post-load hyperinsulinemia, with limited ability to distinguish between complication phenotypes.

### 3.4. Exploratory Anthropometric, Inflammatory, and Biochemical Findings

In addition to glycemic outcomes, several anthropometric, inflammatory, and biochemical parameters were examined as exploratory secondary findings to provide context regarding multisystem involvement. Participants with arterial hypertension had significantly higher body weight (118.50 ± 22.58 kg vs. 99.42 ± 21.46 kg, *p* = 0.005) and higher BMI (40.90 ± 7.75 kg/m^2^ vs. 34.59 ± 5.62 kg/m^2^, *p* = 0.002) compared with those without hypertension. Hematocrit values were higher in participants with hypertension (43.76 ± 4.03% vs. 40.43 ± 3.33%, *p* = 0.005), whereas erythrocyte sedimentation rate values were lower (9.74 ± 7.30 mm/h vs. 15.88 ± 9.99 mm/h, *p* = 0.037). Participants with gallstones exhibited higher platelet counts (315.25 ± 32.42 × 10^3^/μL vs. 263.23 ± 52.93 × 10^3^/μL, *p* = 0.034) and higher CRP concentrations (6.22 ± 4.37 mg/L vs. 3.20 ± 6.99 mg/L, *p* = 0.033) compared with those without gallstones. In participants with OHS or COPD, γ-glutamyltransferase levels were higher (31.18 ± 19.35 U/L vs. 19.20 ± 7.83 U/L, *p* = 0.009), and fibrosis score values differed significantly compared with those without respiratory complications (*p* = 0.007).

## 4. Discussion

The present study provides evidence that, in people living with obesity without T2DM, elevated 1 h post-load plasma glucose during OGTT is consistently associated with a wide range of obesity-related complications involving multiple organ systems. Importantly, these associations were identified at first clinical presentation and in the absence of overt diabetes, indicating that exaggerated early post-load glycemic excursions are associated with a level of metabolic and systemic dysregulation that is already clinically apparent. The novelty of this work lies in highlighting 1 h post-load glucose not only as a marker of future dysglycemia but also as a concurrent marker associated with multisystem involvement in obesity. It should be acknowledged, however, that higher 1 h post-load glucose values may reflect broader cardiometabolic risk clustering rather than a direct or isolated contribution of post-load glycemia to individual complications.

The clinical relevance of obesity-related complications extends beyond their prognostic significance, as they increasingly guide therapeutic decision-making. Contemporary management frameworks proposed by the European Association for the Study of Obesity emphasize a complication-driven approach, whereby treatment intensity and modality are tailored according to the presence and severity of obesity-related complications rather than BMI alone [16]. Within this paradigm, early identification of individuals with a high complication burden is critical. The present findings suggest that 1 h post-load glucose may serve as a practical and informative marker to support this approach by identifying individuals living with obesity who already exhibit multisystem involvement and who may warrant closer clinical assessment and consideration of therapeutic strategies guided by overall complication burden.

A central finding of this study is that 1 h post-load glucose demonstrated superior discriminatory capacity compared with fasting and 2 h glucose values across most complication categories. This observation suggests that the early post-load period represents a sensitive phase for detecting metabolic dysregulation. During this phase, glucose homeostasis depends on the coordinated response of insulin secretion, hepatic glucose suppression, peripheral glucose uptake, and insulin clearance [17]. Disruption of these processes results in exaggerated glucose excursions that are most evident at the 1 h time point. Consequently, elevated 1 h post-load glucose likely reflects the integrated manifestation of multiple disturbed regulatory processes, rather than a single defect, explaining its strong association with diverse obesity-related complications [18].

The importance of 1 h post-load glucose is further supported by extensive evidence from diabetes research. Elevated 1 h post-load glucose during OGTT has been consistently associated with progression to T2DM, cardiovascular events, microvascular complications, and increased mortality, even among individuals with normal fasting and 2 h glucose levels [19]. These observations have positioned 1 h post-load glucose as a sensitive marker associated with early β-cell stress and impaired metabolic flexibility. The present study extends this concept to obesity, demonstrating that the same glycemic abnormality linked to adverse diabetes outcomes is also associated with the presence of obesity-related complications before diabetes onset. This continuity suggests overlapping metabolic disturbances linking early post-load hyperglycemia to both future diabetes risk and current organ dysfunction.

The association between elevated 1 h post-load glucose and complications affecting cardiovascular, renal, respiratory, and functional domains highlights the potential role of early glycemic excursions as markers associated with processes linked to tissue-level injury. Repeated postprandial glucose spikes have been shown to induce oxidative stress, endothelial dysfunction, inflammatory activation, and neurohormonal dysregulation, mechanisms that may contribute to vascular stiffness, renal microvascular damage, and cardiometabolic burden independently of sustained hyperglycemia [20,21,22,23,24,25]. In this context, 1 h post-load glucose may act as a surrogate marker of cumulative glycemic stress within a broader cardiometabolic risk profile that is not adequately captured by fasting or late post-load measurements. The additional biochemical findings reported in this study should be interpreted as supportive indicators of systemic involvement rather than as primary outcomes, and they do not modify the central glycemic associations observed.

Insulin measurements during OGTT provided important complementary insights but showed limited discriminatory capacity for obesity-related complications. The pronounced post-load hyperinsulinemia observed during OGTT, with peak insulin concentrations at approximately 1 h and sustained elevation thereafter, is consistent with a compensatory insulin response to reduced peripheral insulin sensitivity, reflecting insulin resistance as a generalized metabolic background feature in this cohort with obesity. Notably, the absence of a statistically significant decline in insulin concentrations between 1 and 2 h after glucose ingestion suggests impaired metabolic flexibility, with prolonged compensatory insulin exposure rather than efficient post-load recovery. Only in arterial hypertension was prolonged post-load hyperinsulinemia observed, suggesting delayed insulin clearance or sustained compensatory secretion. Overall, these findings indicate that insulin resistance alone does not explain heterogeneity in obesity-related complications, whereas 1 h post-load glucose appears to be more closely associated with downstream metabolic and systemic manifestations of impaired glucose regulation [26,27,28,29,30,31].

Several strengths of this study should be emphasized. Participants were evaluated during their first visit to obesity clinics, prior to exposure to anti-obesity pharmacotherapy or structured weight-loss interventions, minimizing treatment-related confounding. The comprehensive assessment of a broad spectrum of obesity-related complications allowed evaluation of multisystem involvement within a single cohort. Additionally, the use of repeated glucose and insulin measurements during OGTT, analyzed with appropriate non-parametric longitudinal methods, enabled robust characterization of post-load metabolic dynamics in a real-world population without diabetes.

Some limitations must also be acknowledged. The cross-sectional design and retrospective recording of obesity-related complications preclude causal inference, and the modest sample size limited statistical power for less prevalent conditions. Importantly, obesity-related complications were recorded retrospectively and were not defined within a predefined causal or temporal framework. As a result, several cardiometabolic variables closely linked to obesity, including hypertension, dyslipidemia, and systemic inflammation, may represent shared manifestations of underlying metabolic dysfunction rather than independent confounders. Under these conditions, formal multivariable modeling would be susceptible to overadjustment or collider bias and could yield misleading estimates of independent associations. Therefore, potential confounding by coexisting cardiometabolic risk factors could not be formally accounted for in the present analyses. In addition, residual confounding from unmeasured factors such as diet, physical activity, smoking status, medication use, or genetic susceptibility cannot be excluded. Because complication assessment was clinically driven rather than systematic, certain conditions—particularly those requiring imaging or specialized testing—may have been underdiagnosed or misclassified. This potential misclassification or underdiagnosis represents a source of information bias and may have attenuated between-group differences, leading to conservative estimates of the associations observed. Finally, statistically significant differences observed in low-prevalence complication categories should be considered hypothesis-generating and require confirmation in larger cohorts. Nevertheless, the consistency of higher 1 h post-load glucose values across multiple, distinct complication categories supports the biological plausibility and potential clinical relevance of the findings.

## 5. Conclusions

In people living with obesity without diabetes, higher 1 h post-load plasma glucose concentrations during OGTT were consistently observed in individuals with a greater burden of multisystem obesity-related complications. These findings highlight the potential clinical relevance of 1 h post-load glucose as a marker associated with early metabolic and systemic dysregulation in obesity. However, given the cross-sectional design and the retrospective recording of complications, causal relationships cannot be established, and the independent contribution of post-load glycemia cannot be disentangled from coexisting cardiometabolic risk factors. Future prospective studies are therefore warranted to clarify the role of 1 h post-load glucose within the broader context of cardiometabolic risk clustering and to determine whether it may meaningfully inform complication-driven risk stratification within a broader cardiometabolic risk assessment framework.

## Figures and Tables

**Table 1:** Baseline demographic, anthropometric, metabolic characteristics and prevalence of obesity-related complications (*n* = 47).

Variable	Value
Age (years)	47.53 ± 11.29
Height (m)	1.69 ± 0.09
Body weight (kg)	106.73 ± 23.59
Body mass index (kg/m^2^)	37.01 ± 7.14
Waist circumference (cm)	113.38 ± 16.80
Waist-to-height ratio	0.67 ± 0.11
Fasting glucose (mg/dL)	102.07 ± 20.02
Fasting insulin (μIU/mL)	24.30 ± 27.80
1 h post-load glucose (mg/dL)	156.91 ± 56.24
2 h post-load glucose (mg/dL)	108.46 ± 45.23
Arterial hypertension	18 (38.3%)
Dyslipidemia	30 (63.8%)
MASLD	29 (61.7%)
Impaired glucose tolerance	20 (42.6%)
OHS/COPD	26 (55.3%)
Obstructive sleep apnea	15 (31.9%)
Urinary incontinence	4 (8.5%)
Gallstones	4 (8.5%)
Chronic kidney disease	2 (4.3%)
ASCVD	2 (4.3%)

Abbreviations: MASLD, metabolic dysfunction-associated steatotic liver disease; OHS, obesity hypoventilation syndrome; COPD, chronic obstructive pulmonary disease; ASCVD, atherosclerotic cardiovascular disease.

**Table 2:** Differences in 1 h post-load glucose according to obesity-related complications.

Complication	Absence of Complication	Presence of Complication	*p*-Value
Arterial hypertension	132.38 ± 35.70	196.44 ± 61.46	<0.001
Dyslipidemia	130.24 ± 37.35	172.03 ± 59.95	0.020
MASLD	127.05 ± 37.48	175.45 ± 58.43	0.005
Impaired glucose tolerance	140.85 ± 46.43	178.60 ± 62.02	0.027
OHS/COPD	139.14 ± 40.22	171.27 ± 63.61	0.039
Urinary incontinence	152.42 ± 55.72	205.25 ± 40.31	0.038
Chronic kidney disease	154.38 ± 56.14	214.00 ± 2.83	0.048
Polycystic ovary syndrome	155.98 ± 55.41	160.33 ± 59.27	0.742

Values are presented as mean ± standard deviation (mg/dL). All values represent unadjusted between-group comparisons. No multivariable analyses were performed due to the retrospective recording of complications and the exploratory nature of the study. Abbreviations: MASLD, metabolic dysfunction-associated steatotic liver disease; OHS, obesity hypoventilation syndrome; COPD, chronic obstructive pulmonary disease.

## Data Availability

The data presented in the study are available upon request from the corresponding author. The data are not publicly available due to privacy restrictions of the Greek National Health System.
